# Dynamic Modelling under Uncertainty: The Case of *Trypanosoma brucei* Energy Metabolism

**DOI:** 10.1371/journal.pcbi.1002352

**Published:** 2012-01-19

**Authors:** Fiona Achcar, Eduard J. Kerkhoven, The SilicoTryp Consortium, Barbara M. Bakker, Michael P. Barrett, Rainer Breitling

**Affiliations:** 1 Institute of Molecular, Cell and Systems Biology, College of Medical, Veterinary and Life Sciences, University of Glasgow, Glasgow, United Kingdom; 2 Groningen Bioinformatics Centre, Groningen Biomolecular Sciences and Biotechnology Institute, University of Groningen, Groningen, The Netherlands; 3 Wellcome Trust Centre for Molecular Parasitology, Institute of Infection, Immunity and Inflammation, College of Medical, Veterinary and Life Sciences, University of Glasgow, Glasgow, United Kingdom; 4 Department of Liver, Digestive and Metabolic Diseases, University Medical Centre Groningen, University of Groningen, The Netherlands; University of Virginia, United States of America

## Abstract

Kinetic models of metabolism require detailed knowledge of kinetic parameters. However, due to measurement errors or lack of data this knowledge is often uncertain. The model of glycolysis in the parasitic protozoan *Trypanosoma brucei* is a particularly well analysed example of a quantitative metabolic model, but so far it has been studied with a fixed set of parameters only. Here we evaluate the effect of parameter uncertainty. In order to define probability distributions for each parameter, information about the experimental sources and confidence intervals for all parameters were collected. We created a wiki-based website dedicated to the detailed documentation of this information: the SilicoTryp wiki (http://silicotryp.ibls.gla.ac.uk/wiki/Glycolysis). Using information collected in the wiki, we then assigned probability distributions to all parameters of the model. This allowed us to sample sets of alternative models, accurately representing our degree of uncertainty. Some properties of the model, such as the repartition of the glycolytic flux between the glycerol and pyruvate producing branches, are robust to these uncertainties. However, our analysis also allowed us to identify fragilities of the model leading to the accumulation of 3-phosphoglycerate and/or pyruvate. The analysis of the control coefficients revealed the importance of taking into account the uncertainties about the parameters, as the ranking of the reactions can be greatly affected. This work will now form the basis for a comprehensive Bayesian analysis and extension of the model considering alternative topologies.

## Author Summary

An increasing number of mathematical models are being built and analysed in order to obtain a better understanding of specific biological systems. These quantitative models contain parameters that need to be measured or estimated. Because of experimental errors or lack of data, our knowledge about these parameters is uncertain. Our work explores the effect of including these uncertainties in model analysis. Therefore, we studied a particularly well curated model of the energy metabolism of the parasite *Trypanosoma brucei*, responsible for African sleeping sickness. We first collected all the information we could find about how the model parameters were defined on a website, the SilicoTryp wiki (http:///silicotryp.ibls.gla.ac.uk/wiki/). From this information, we were able to quantify our uncertainty about each parameter, thus allowing us to analyse the model while explicitly taking these uncertainties into account. We found that, even though the model was well-defined and most of its parameters were experimentally measured, taking into account the remaining uncertainty allows us to gain more insight into model behavior. We were able to identify previously unrecognised fragilities of the model, leading to new hypotheses amenable to experimental testing.

## Introduction

Kinetic models of metabolism require quantitative knowledge of detailed kinetic parameters (e.g. maximum reaction rates, enzyme affinities for substrates and regulators). However, our knowledge about these parameters is often uncertain. When the parameters are measured, various sources of error can affect the results: experimental noise at the technical and biological levels, systematic bias introduced by parameters being measured *in vitro* instead of *in vivo* or by the choice of specific experimental conditions (pH, temperature, ionic strength, etc.). Moreover, a substantial number of important parameters have never been measured and the estimates included in models are based either on values measured in closely related species or on the general distribution of similar parameters [1]. Few general methods for dealing with this uncertainty have been suggested [2]–[6].

**Figure 1:**
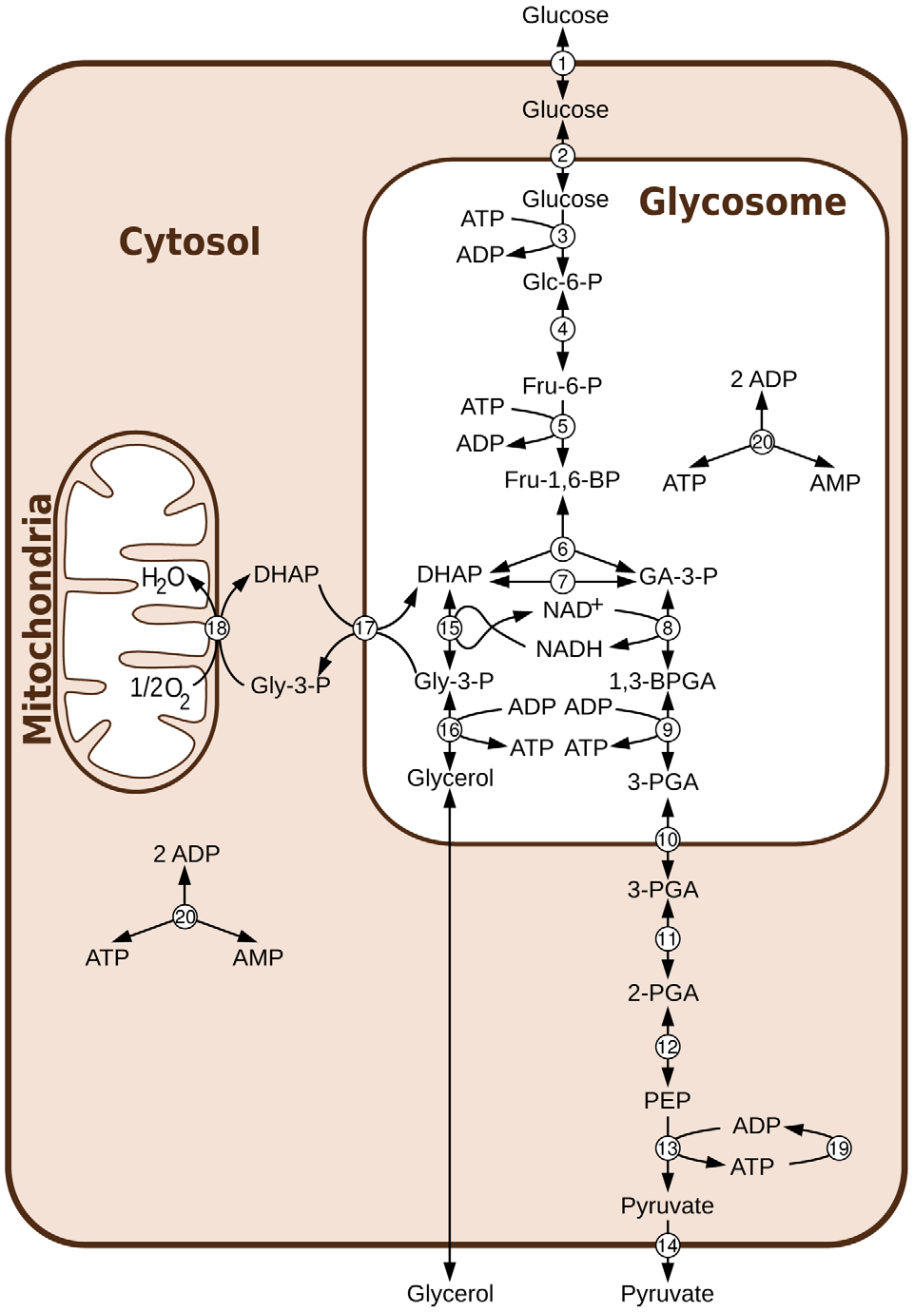
Aerobic glycolysis in bloodstream form *T. brucei*. Abbreviations: Metabolites: Glc-6-P = Glucose 6-phosphate, Fru-6-P = Fructose 6-phosphate, Fru-1,6-BP = Fructose 1,6-bisphosphate, DHAP = dihydroxyacetone phosphate, GA-3-P = glyceraldehyde 3-phosphate, Gly-3-P = glycerol 3-phosphate, 1,3-BPGA = 1,3-bisphosphoglycerate, 3-PGA = 3-phosphoglycerate, 2-PGA = 2-phosphoglycerate, PEP = phosphoenolpyruvate. Reactions: 1 = transport of glucose across the cytosolic membrane, 2 = transport of glucose across the glycosomal membrane, 3 = hexokinase, 4 = phosphoglucose isomerase, 5 = phosphofructokinase, 6 = aldolase, 7 = triosephosphate isomerase, 8 = glyceraldehyde 3-phosphate dehydrogenase, 9 = phosphoglycerate kinase, 10 = transport of 3-PGA across the glycosomal membrane, 11 = phosphoglycerate mutase, 12 = enolase, 13 = pyruvate kinase, 14 = transport of pyruvate across the cytosolic membrane, 15 = glycerol 3-phosphate dehydrogenase, 16 = glycerol kinase, 17 = DHAP-Gly-3-P antiporter, 18 = glycerol-3-phosphate oxidation, 19 = ATP utilisation, 20 = adenylate kinase.

Here we present an analysis of the effect of parameter uncertainties on a particularly well defined example of a quantitative metabolic model: the model of glycolysis in bloodstream form *Trypanosoma brucei* [7] (see Fig. 1). This ordinary differential equation (ODE) model is mainly using parameters measured on purified enzymes rather than fitted, and, since its first publication in 1997, it has been updated [8] and extended [9]–[11] several times, making it one of the most highly refined dynamic models of a metabolic pathway published to date. The model has been successfully used to predict the “turbo explosion” that would happen in the absence of the glycosome, the subcellular compartment in which the first seven enzymes of glycolysis are localized in *T. brucei* [8]. This important property was confirmed experimentally more than 10 years after the model was initially proposed [11]. In this paper we used the last updated version of the model published [11] with slight modifications to take into account the equilibrium constants of all reactions (see methods).

Explicitly considering the uncertainties of parameters in the analysis of the model allowed us to gain interesting new insights into its behaviour. Most importantly, our analysis allowed us to quantify the degree of confidence concerning diverse properties of the system, including the hierarchy of control which is relevant for prioritizing potential drug targets. The resulting quantitative profile of model uncertainties, including the identification of major fragilities and areas in need of further examination, provides a solid basis for future model extensions. These will in turn introduce new uncertainties and should be dealt with using the same general framework established here.

## Results

### Collecting information

**Figure 2:**
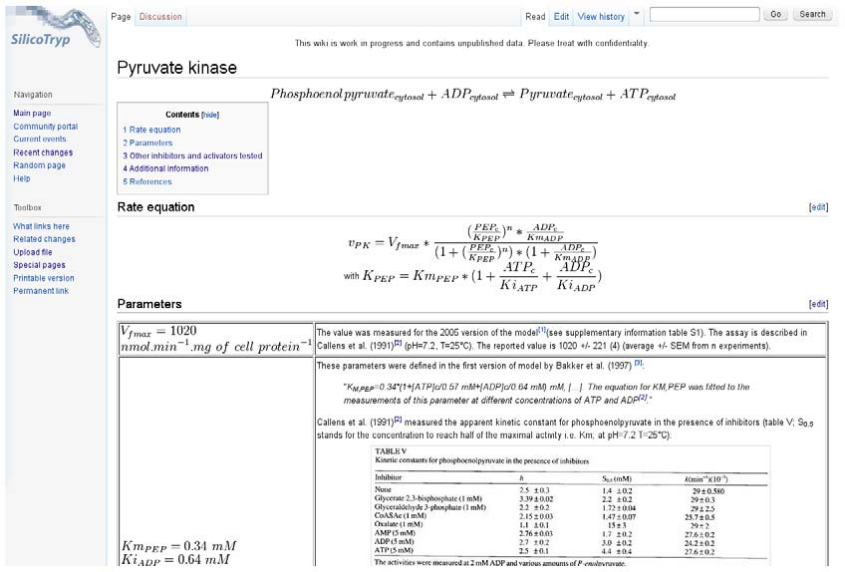
Example of a page of the SilicoTryp wiki. Each reaction of the model has its own page. On this page, the rate equation is specified and a table includes all parameters with their detailed source and calculations when necessary.

In order to specify the uncertainty associated with each parameter, we gathered all available information relating to the sources of the values used in the model. Information included data on how kinetics were measured, the number of replicates and the standard error of mean values when available, additional calculations used to estimate the parameter from the observed values, and any “corrections” for additional factors such as temperature or pH. For this purpose, we created the “SilicoTryp” wiki, a MediaWiki-based (http://www.wikimedia.org) website dedicated to the detailed documentation of the sources of parameters used in the latest version of the model of glycolysis in *T. brucei* (http://silicotryp.ibls.gla.ac.uk/wiki/Glycolysis) [10]. Each reaction is described on its own page, which contains the rate equation and the detailed references and calculations for each parameter (see Fig. 2 for an example).

From the information collected, probability distributions could be inferred for each parameter as described in Methods. supplementary text S1 shows the estimated distributions for all parameters.

### The effects of uncertainty

To model the effect of uncertainty, we sampled values for each parameter according to its probability distribution, generating a ensemble of alternative models. Together these alternative models accurately represent our degree of uncertainty about the correct parameters, assuming that our knowledge of each parameter value is independent of the other parameters (see Methods for one example, the equilibrium constant, where this assumption is violated and needs to be accounted for). This collection of models can then be used to analyse model behavior and the associated uncertainties. The same properties that were studied with the fixed parameter version of the model can be studied with each alternative model. The distribution of the results shows the robustness and the degree of certainty we have about the inferred model properties (e.g. the steady-state concentrations of the metabolites and the control coefficients) considering our current knowledge about the parameters and the topology of the model.

#### Reaching steady-state

**Figure 3:**
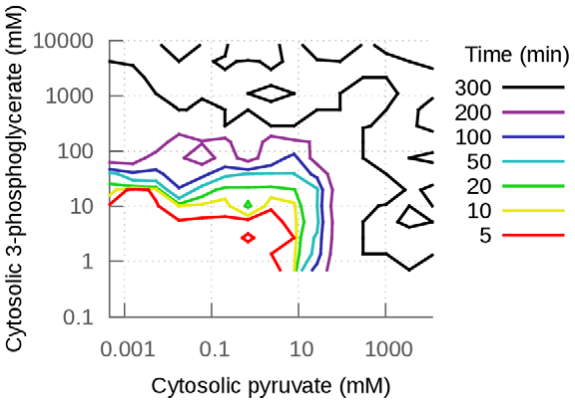
Steady-state concentration of pyruvate as a function of the concentration of 3-phosphoglycerate at steady-state or t = 300 minutes if steady-state is not reached before. The contour lines indicate when steady-state was reached (in minutes of simulated time). If steady-state was not reached before, simulations were stopped at 300 minutes (see Methods). When a model did not reach steady-state before 300 minutes, the concentrations of pyruvate and/or 3-phosphoglycerate reached unreasonably high concentrations (black contour lines). Note that the models that do not reach steady-state within 300 minutes because of 3-PGA accumulation will eventually reach steady-state at very high 3-PGA concentrations if the simulations are run much longer. This is not the case for the models that show pyruvate accumulation. Since pyruvate kinase is not product-sensitive in the model, nothing stops the accumulation of pyruvate and steady state is never reached (see supplementary Fig. S1 for example of simulations).

The first property of the models that we analyzed is whether or not a steady-state is reached in a reasonable time. Our simulation uses the steady-state of the model with the fixed set of parameters to set the initial concentrations of the metabolites. From this initial state, each model is simulated until steady-state is reached. Considering the generous threshold we set for these simulations, steady-state should be reached rapidly. Yet, only 33% of the 10,000 models reached steady-state within 50 simulated minutes or less, and only 36% within 300 simulated minutes. As shown in Fig. 3, models that could not reach steady-state within 300 minutes had all produced a very high concentration of either 3-phosphoglycerate (3-PGA) or pyruvate. The accumulation of these metabolites to unreasonable concentrations indicates that the models contain fragilities. These cases are studied in more detail below (section Effects on steady-state concentrations).

#### Effects on steady-state flux

In bloodstream form *T. brucei*, glucose is mainly converted to pyruvate in aerobic conditions, while it is divided equally between pyruvate production and glycerol production under anaerobic conditions [12], [13]. These flux distributions were also observed in cultured cell [14] and reproduced by the model [7] (anaerobic conditions are modelled by setting the 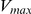 of glycerol 3-phosphate oxidase (GPO, reaction used to model the mitochondrial glycerol 3-phosphate dehydrogenase coupled with the trypanosome alternative oxidase) to zero, the only model reaction requiring oxygen; the experimental measurements correspond to its inhibition as *T. brucei* does not survive total anaerobic conditions [9]).

**Figure 4:**
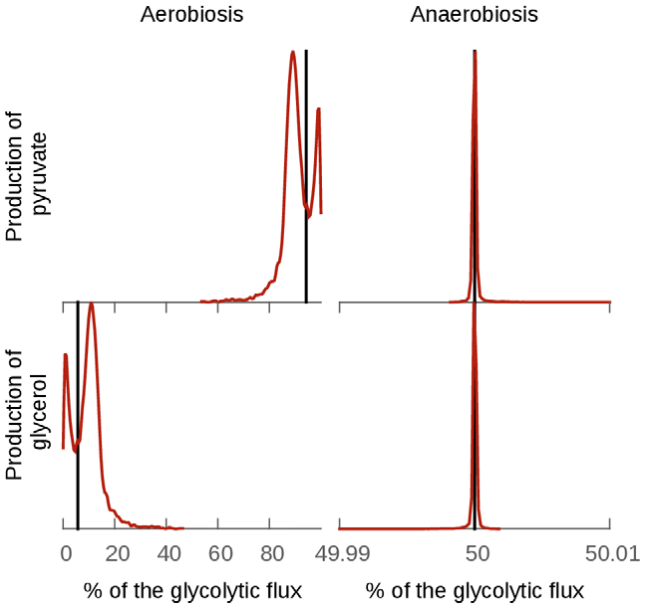
Effect of the uncertainties on the distribution of the glycolytic flux between the production of pyruvate and glycerol. The glycolytic flux is defined as the sum of the fluxes producing glycerol and pyruvate. The black lines represents the percentage of the glycolytic flux in the pyruvate branch (top) and the glycerol branch (bottom) in the fixed parameter model. The red line is the distribution of the percentage of the glycolytic flux in the collection of models generated from the parameter probability distributions. The division of the flux between the pyruvate branch and the glycerol branch is well conserved. The effect of the uncertainties of the parameters is almost non-existent in anaerobic conditions (simulated by setting the glycerol 3-phosphate oxidase 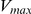 parameter to 0). In aerobic conditions the effect is more important, indicating that this division is not entirely due to the topology of the model in this case.

This property is well-conserved in all our models using the full range of plausible parameter values (see Fig. 4). As expected, the effect of uncertainty is more important in aerobic conditions: for most of the models that do reach steady-state within 300 minutes, the proportion of glucose that ends in glycerol varies between 0 and 20% (mean 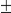 standard deviation of the models that reaches steady-state within 300 minutes: 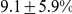). In contrast, under anaerobic conditions, the glycolytic flux is always shared 50/50% between the production of glycerol and pyruvate (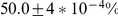; the small error is most probably due to numerical rounding effects).

Indeed, anaerobically, the flux distribution is entirely determined by the topology and stoichiometry of the model: the 6-carbon product derived from glucose (fructose 1,6-bisphosphate) is split into two 3-carbon products by aldolase. Anaerobically, the NADH formed in the pyruvate branch can only be reoxidized to 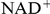 in the glycerol branch [7]. Hence the 50/50% split is independent of the parameters of the model as expected from the topology of the model. Under aerobic conditions, glycerol 3-phosphate is mostly reoxidized using the mitochondrial glycerol 3-phosphate dehydrogenase (GPO) and then re-routed through the pyruvate branch via triose-phosphate isomerase [7]. However, a small proportion of the flux ends with the production of glycerol. This small proportion depends on the parameters used in the model. No single parameter can easily predict the proportion of the flux that ends in the production of glycerol.

The fixed-parameter version of the model (i.e. the model with the set of parameter defined to be as close as possible to the model described in [10]) predicted only a very small portion of the glycolytic flux through the glycerol branch, while a wider range of plausible values are permitted when parameter uncertainty is considered. Indeed, a wide range of aerobic flux distributions has been measured experimentally: from a few percent glycerol measured by [14] to about 9% glycerol measured by [15]. This range of observed biological variety can be explained using the variety of kinetic parameters included in our collection of models, or by partial anaerobiosis leading to a mixture of oxygenation states in individual cells within the population measured.

#### Effects on steady-state concentrations

**Figure 5:**
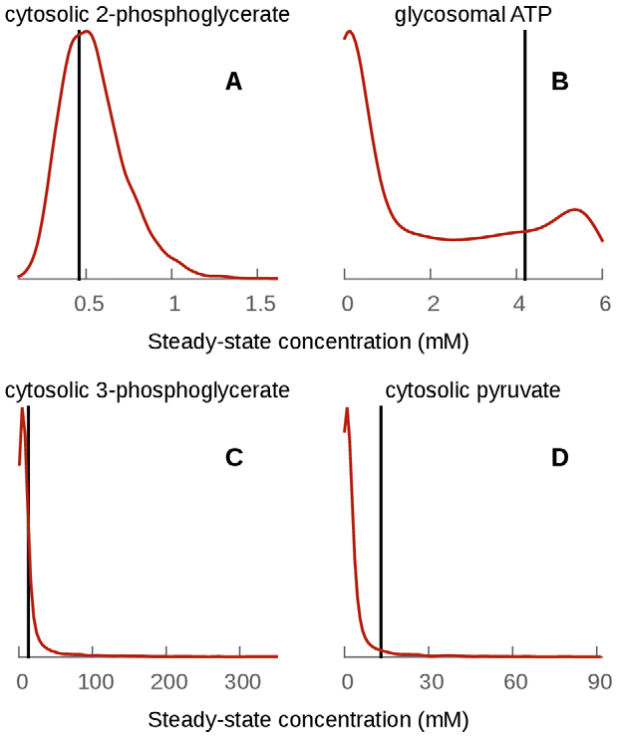
Distribution of the steady-state concentrations of four metabolites. The cytosolic 2-phosphoglycerate and glycosomal ATP steady-state concentrations are consistent with physiological metabolite concentration, whereas 3-phosphoglycerate and pyruvate sometimes reach hundreds of millimoles per liter. The value for the fixed parameter model is indicated by a vertical black line.

Using our collection of models, we are able to see the effect of parameter uncertainties on the steady-state concentration estimates. Considering only the models that reach steady-state within 300 simulated minutes, several cases can be distinguished (see Fig. 5 and supplementary text S2):

- For many metabolites, steady-state concentrations are well-conserved in all plausible models and their distribution is approximately log-normal: glucose 6-phosphate, fructose 6-phosphate, glycosomal glyceraldehyde 3-phosphate, cytosolic and glycosomal dihydroxyacetone phosphate and glycerol 3-phosphate, 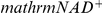, NADH, 2-phosphoglycerate and phosphoenolpyruvate. These distributions may be expected, given that most of the parameters are sampled from log-normal distributions and thus approximately log-normal distributions for the steady-state concentrations are expected too.
- For several metabolites, steady-state concentrations do not follow approximate log-normal distributions, although their steady-state concentrations are distributed within a range of values consistent with physiological metabolite concentrations. These include glycosomal and cytosolic ATP, ADP, AMP, glycosomal and cytosolic glucose, fructose 1,6-bisphosphate and glycosomal 1,3-bisphosphoglycerate. For example, the concentration of glycosomal ATP and AMP is predicted to be between 0 and 6 mM (see Fig. 5 B. for ATP), compared to the fixed-parameter values of 4.2 mM and 0.25 mM respectively. The concentration is bounded by the fact that the total adenine nucleotide concentration in the glycosome is set to 6 mM in the model. Given the uncertainty regarding the exact parameters, any ratio between ATP and AMP seems possible and consistent with our parameter knowledge.
- For two metabolites, 3-phosphoglycerate and pyruvate, the steady-state concentration distribution has a long, heavy tail, indicating that some combinations of plausible parameter values can lead to extreme predicted concentrations (several hundreds to thousands of 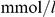, see Fig. 3). These cases were studied in more detail as they point to interesting fragilities in the existing model, which indicate a need to refine our knowledge of some parameters and/or model topology.

**Figure 6:**
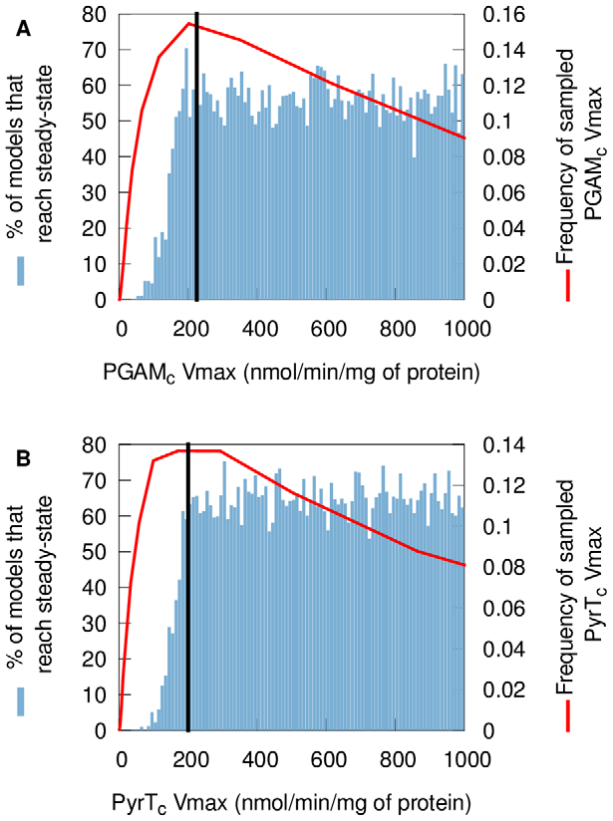
Percentage of sampled models that reach steady-state within 300 minutes as a function of the 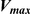 of pyruvate transport and phosphoglycerate mutase. (A) Percentage of models that reach steady-state within 300 minutes as a function of phosphoglycerate mutase 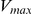 (B) Percentage of models that reach steady-state within 300 minutes as a function of pyruvate transport 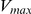. The red line is the distribution of the parameter as it is usually sampled. The black line is the fixed-parameter value. A model which has a value for one of these two parameters smaller than the mean will easily fail to reach steady-state, whatever the other parameter values and despite these 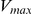 values still being close to their mean. This reveals fragilities in the model.

The accumulation of 3-phosphoglycerate (3-PGA) and/or pyruvate to unreasonable concentrations causes some models to reach steady-state at extremely high concentrations or to fail reaching steady-state within 300 minutes. This occurs when the maximum reaction rates (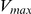) of phosphoglycerate mutase (PGAM) for the 3-PGA accumulation or pyruvate transport (PyrT) for the pyruvate accumulation are smaller than their mean values. Fig. 6 shows the percentage of models that break as a function of PGAM 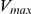 (Fig. 6 A) and PyrT 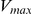 (Fig. 6 B). The data show that these models break even when these parameters have values very close to the original value used in the fixed-parameter version of the model. Yet, these two reactions can both be inhibited experimentally *in vivo*. When PGAM was inhibited using tetracycline-inducible RNAi, diminishing 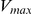 to 51% of its original value [10], no adverse effects on the viability of the organism were observed. The pyruvate transporter can also be inhibited substantially before the cells start dying [16]. The reason for the newly revealed model fragilities thus could be twofold: either the relevant parameter values are significantly higher than the currently used values (which are fitted, not measured; [10]), or some unknown regulatory interaction or missing reaction stabilizes the biological system. The pyruvate accumulation is due to a known fragility of the model: the pyruvate kinase is insensitive to its products, which can lead to the accumulation of pyruvate when the 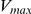 of its transporter is not high enough.

Vanderheyden et al. have measured the pyruvate efflux 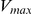 at 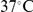 in bloodstream form *T. brucei* [17] as 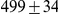 nmol/min/mg of protein (mean 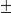 SD). Assuming an activation energy of 50 kJ/mol, the 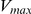 at 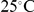 would be about 230 nmol/min/mg of protein, very close to the 200 nmol/min/mg of protein currently used in the model. Using Vanderheyden et al.'s value to compute the probability distribution of the 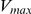 of pyruvate transport (using the corrected mean and calculating the standard deviation as for any value with a measured mean and unknown standard deviation; see methods), only 0.58% of models cannot rapidly reach a steady-state because of pyruvate accumulation (pyruvate concentration at 300 minutes higher than 100 mM), compared to 35.4% when the 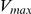 is set as described in Methods). But this still imply that even a small inhibition of the pyruvate transporter should kill the cells, which is inconsistent with the experimental observations [16]. Therefore, there is probably an additional mechanism that prevents the accumulation of pyruvate in the cytosol. Among the possible hypotheses, it is interesting to note that alanine aminotransferase activity has been measured in bloodstream forms by Spitznagel et al. [18] (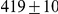 nmol/min/mg of protein in whole cell extracts at 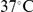). This enzyme, which catalyses the reversible reaction 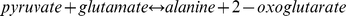, was shown to be essential in bloodstream form trypanosomes and might have a significant role in the regulation of the intracellular pyruvate concentration.

Adding alanine aminotransferase into the model would require adding several other reactions as well: the production and recycling of 2-oxoglutarate and glutamate need to be incorporated, as well as the export of alanine [19], [20] and probably 2-oxoglutarate [21].

#### Effects on control coefficients

Control coefficients are one of the most important high-level properties of kinetic models of metabolism: they allow the quantification of how much influence each reaction has on the flux of the pathway. In the glycolytic model of *T. brucei*, individual control coefficients have been used to predict the most promising trypanocidal drug targets. The *T. brucei* glycolysis model published by Alberts et al. [10] indicated that the glucose consumption flux is controlled mainly by the glucose transporter at 5 mM of extracellular glucose (control coefficient 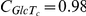 [10]). As the sum of the control coefficients over the pathway is one [22], the other enzymes have no or very little control over the glucose consumption flux in this fixed-parameter model.

**Figure 7:**
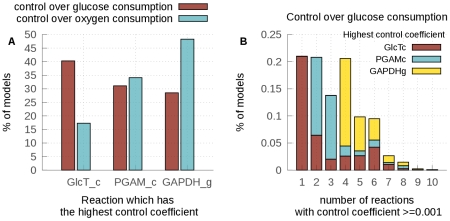
Control coefficients in the collection of models. (A) Percentage of models which have either the glucose transporter (GlcTc), phosphoglycerate mutase (PGAM) or glyceraldehyde 3-phosphate dehydrogenase (GAPDH) as the reaction with the highest control coefficient either over the glucose consumption flux (red) or the oxygen consumption flux (blue). (B) Percentage of models vs. the number of reactions that have a control coefficient higher than 0.001. The color inside the bars represents the proportion that has either the glucose transporter, PGAM or GAPDH as the reaction with the highest control coefficient over the glucose consumption flux within these subgroups.

Using our collection of models, we calculated the control coefficients for every reaction and every model (see Methods). These control coefficients were then ranked from the highest to the lowest. Our analysis (Fig. 7) shows that, given our uncertainty on the parameters, we cannot be certain about the identity of the reaction that has most control over the glucose consumption flux. Moreover, we show that the fixed-parameter model scenario, where almost all the control is held by one reaction - the glucose transporter - is not the only scenario possible, but that even at 5 mM of glucose the control might be shared by several reactions.

Fig. 7A (red) shows the reaction that has the highest control coefficient over the glucose consumption flux as a percentage of the sampled model. The glucose transporter has the highest control over the glucose consumption flux in only 40.3% of the models. A substantial proportion of models yield either the phosphoglycerate mutase (PGAM, 31.1%) or GAPDH (28.5%) as having the highest control coefficient.

In 1999, Bakker et al. [23] estimated the control coefficient over the oxygen consumption flux of the glucose transporter experimentally (at 5 mM of extracellular glucose) as being between 0.3 and 0.5. In the fixed-parameter model, the 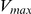 of the glucose transporter was fitted to this control coefficient (0.4) by Alberts et al. [10]. In 17.3% of our models, the glucose transporter has the highest control coefficient over the oxygen consumption flux (Fig. 7A (blue)). Among these models, the control coefficient of the glucose transporter varies between 0.2 and 1.0; when another reaction has the largest control coefficient, the control coefficient of the glucose transporter is always lower than 0.4. A similar distribution of the control coefficient is observed over the glucose consumption flux. No single parameter alone can explain the wide range of values of 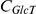. It has been shown, however, that the extreme sensitivity of 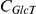 to various parameters can be attributed to the large difference between the 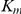 values of the glucose transporter and the next enzyme, hexokinase, towards intracellular glucose [24].

Fig. 7B represents the number of reactions that exert some control over the glucose consumption flux (defined as the reactions with control coefficients above 0.001) as a percentage of the sampled models. For 21% of the sampled models, only one reaction controls the glucose consumption flux and this reaction is most of the time the glucose transporter, as is the case in the fixed-parameter model. When PGAM exerts most control over the flux, it shares the control with at least one other reaction. When GAPDH exerts most control over the flux, it shares the control with at least four other reactions. Interestingly, the activity of GAPDH has been reported to be inhibited by an unknown compound [25]. If this inhibition is physiological and also occurs *in vivo*, it might have an important role in the control of glycolytic flux. Indeed, partial inhibition of GAPDH has already been shown to decrease the glycolytic flux and kill the cell [26]. The fact that PGAM exerts the most control in some models might reflect our lack of knowledge about the parameters describing the rate of this reaction. Further analysis of the kinetics of this reaction is necessary to know whether it really exerts some control over the glycolytic flux and thus represents interesting potential drug target.

In the fixed-parameter model, shared control was only seen at glucose concentrations higher than 5 mM [8]. Taking our uncertainty about the parameters values into account shows that this might be the case already at 5 mM of glucose, as has already been suggested by the preliminary analysis of the sensitivity of control coefficients to variations in 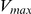 values in this model [8]. Improving our knowledge about GAPDH and PGAM parameters will allow us to know with a higher degree of confidence if only one of these scenarios is relevant *in vivo* or if a similar diversity can be found in a parasite population. This knowledge will be essential to predict if a single glycolytic drug target is sufficient or if multiple reactions need to be inhibited to control parasite infections.

## Discussion

Dynamic models of metabolism are powerful tools to infer interesting and often unexpected properties of cellular physiology. However, the data used to build models from diverse sources can lack accuracy and precision. Here we demonstrate how model output can vary when the uncertainties associated with incomplete and variable datasets are explicitly considered in studying a model. We took as an example the well characterised model of the compartmentalised glycolysis in the parasitic protozoan *T. brucei*. It should be noted that our assessment of the effect of parameter uncertainty on the conclusions that are possible is very conservative. Whenever possible, we have restricted our uncertainty estimates to the level of experimental uncertainty seen within a single assay. This ignores the systematic effects of differences in, e.g., temperature, pH or ion compositions, or biases introduced in sample preparation, all of which would increase uncertainty as can also be seen when parameter values from different laboratories are compared. However, even with these relatively limited uncertainties, we were able to assess the robustness and variability of various properties of the model.

The first property that we studied is the ability of the model to reach steady-state rapidly. Surprisingly, a significant proportion (60%) of the models we generated by sampling the parameters did not allow the model to reach steady-state within 300 minutes, due to the accumulation of either 3-phosphoglycerate or pyruvate in the cytosol. This phenomenon could be attributed to two individual parameters, the maximal reaction rates of phosphoglycerate mutase and pyruvate transport which, when operating below their mean value (but still very close to it), caused the accumulation of two metabolites (3-phosphoglycerate and pyruvate respectively). For the pyruvate transporter, the analysis suggested a mechanism that could avoid this problem: alanine aminotransferase has been shown, unexpectedly, to be essential in bloodstream form *T. brucei* [18], and its activity comparable with the rate of pyruvate efflux. This would be sufficient to exert a substantial influence on the intracellular pyruvate concentration. The maximal reaction rate of phosphoglycerate mutase is difficult to measure directly [27], therefore further experimental and theoretical studies are required to refine our knowledge about this reaction. Indeed, the model predicts that current values for PGAM are probably lower than those operative in *T. brucei*, and some effort should be made to determine whether the values are indeed higher.

We then analysed the distribution of the steady-state fluxes between the pyruvate and glycerol producing branches of glycolysis both in aerobic and anaerobic conditions. In totally anaerobic conditions, the distribution was very well conserved. Indeed, this property is entirely constrained by the topology of the model and thus this result was expected. Our analysis shows that the distribution of the fluxes is more variable in aerobic conditions, consistent with previously unexplained variation in experimental observations (although changes in oxygen tension within different cells in measured populations would create the same effect).

Further analysis of the steady-state concentrations allowed us to distinguish the metabolites that are only moderately affected by the parameter uncertainties and follow an approximate log-normal distribution, such as 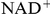 and NADH, from the metabolites that follow a more complex distribution such as glycosomal ATP. ATP is constrained by a conserved sum, therefore its steady-state concentration always stays within reasonable limits. Technical limitations mean that the concentration of glycosomal ATP is not directly accessible for experimentation (glycosomes cannot be purified efficiently enough). Therefore, only by acquiring additional data about the parameters of the model can assumptions about these concentrations at steady-state be refined.

Finally, we analysed the control coefficients of each enzyme using our collection of models. These properties are especially important in the case of glycolysis in *T. brucei*, as they allow us to identify potential drug targets. Our analysis reveals that, although the reaction that has the most control over the glucose consumption flux is the glucose transporter in 40.3% of the models, two other reactions maximally control the flux in a significant proportion of the models: PGAM (31.1%) and GAPDH (28.5%). Moreover, the activity of GAPDH has been reported to be inhibited by an unknown metabolite [25]; if this inhibition occurs *in vivo*, it might have an important role in the control of glycolytic flux. Interestingly, partial inhibition of GAPDH has been shown to affect parasite growth and glycolytic flux [26], and selective inhibitors of the *T. brucei* enzyme have been shown to be trypanocidal [28]. The rest of the control coefficient hierarchy is more variable. Either this variability is a true reflexion of biological noise or the result of our lack of knowledge about some parameters of the model.

The data derived from the work performed here point to several further studies, including analysis of the role of alanine amino transferase in the regulation of pyruvate concentration and more exact quantification of pyruvate transport and phosphoglycerate mutase kinetics. The detailed description of parameter uncertainty will now form the basis for a comprehensive Bayesian analysis and extension of the model using alternative topologies [29]. These analyses will allow us to quantify our posterior belief about the parameters of the model when it is confronted with new experimental data such as measured metabolite concentrations in different conditions.

## Methods

### The model

The model used in this paper is the last updated version [10], [11] of the glycolysis model of *T. brucei* first published by Bakker et al. in 1997 [7] (see Fig. 1).

To allow a straight-forward sampling of parameters, the rate equations were rewritten to contain the equilibrium constant instead of the ratio of 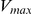 values (reverse over forward), using the Haldane equation [30]. This does not change the rates, but simplifies the sampling of the parameters, as we do not need to check for consistency with the thermodynamic equilibrium constant. For example, the phosphoglucose isomerase (PGI) rate equation was:
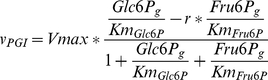
(1)where 
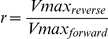
. The Haldane equation gives:
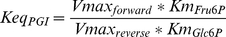
(2)Therefore, the rate equation of PGI can be rewritten as:
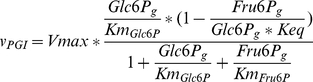
(3)


The list of sources used to compute the values of the equilibrium constants is available in supplementary text S3 and on the SilicoTryp wiki (http://silicotryp.ibls.gla.ac.uk/wiki/Glycolysis). The model in [11] considered the transport reactions between the cytosol and the glycosome and adenylate kinase (see special cases) to be very fast compared the other reactions of the model. Therefore, they were not explicitly modelled. To enable consideration of the effect of parameter uncertainty on the rate of these transport reactions, we modelled them explicitly using mass action kinetics. As we considered that these reactions have an equilibrium constant of unity (no preferential accumulation or exclusion in one of the compartments), we used a single rate parameter for each transport reaction. For example, the transport of glucose between the cytosol and the glycosome is modelled as:
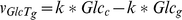
(4)The model is available as supplementary dataset S1 (SBML file [31]). The parameter values are as in [11]. The equilibrium constant are calculated from the 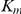 values and the ration of 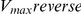 over 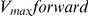 when necessary.

### Probability distributions of the parameters

In order to sample the model parameters, we needed to define a probability distribution for each parameter. These distributions can be defined empirically using arbitrary shapes, but for the sake of convenience it is usually appropriate to use standard shapes (e.g. normal or log-normal distributions) and then to estimate the parameters of these distributions (usually the mean and standard deviation).

#### Km/Ki

These parameters represent concentrations, therefore they cannot be negative and our uncertainty about their values is best represented by a log-normal distribution.

**Figure 8:**
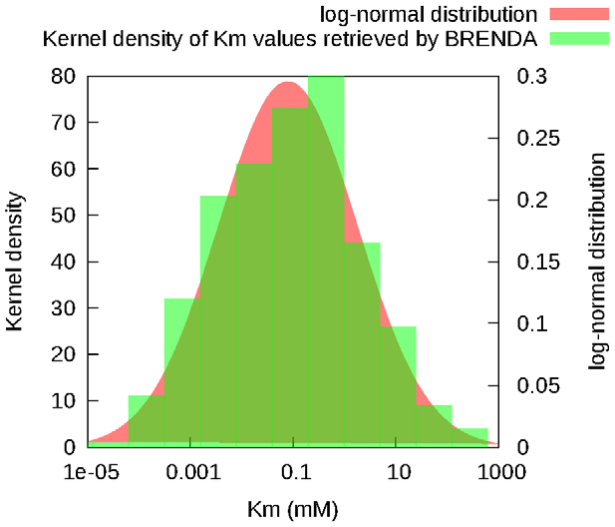
Distribution of the 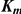 values retrieved from the BRENDA database. 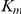
 values retrieved from the database (green) and a log-normal distribution with the same mean and standard-deviation (red) are shown.

For each 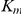 or 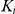 value of the model, the mean and standard deviation of the corresponding log-normal distribution must be estimated from available experimental data (indicated as 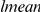 and 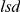). Five situations occur:

- The parameter has been measured experimentally: a mean (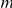) and standard deviation (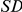) or standard error (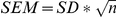, where n is the number of observations) are available. 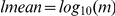 and 
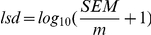
. If a standard deviation is available and the number of observations is not specified, 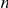 is supposed to be 3.
- The parameter has been measured experimentally, but only a mean value is reported. 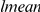 is computed as above, 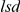 is computed using the average relative standard error (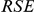) of all 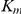 values of the model for which 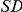 or 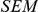 is available. The value of 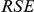 calculated from the published data is usually between 10 and 20%, indicating that the RSE can be expected to be similar for those 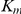 values where it has not been specified.
- The parameter has not been measured, and no estimate of its value is available. When no other information is available, the parameter is calculated from the list of 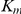 values of all *T. brucei* enzymes retrieved from BRENDA [32] (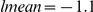, 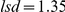, Fig. 8).
- The parameter has not been measured, but some indication of its mean is available, e.g. a value measured for a phylogenetically closely related species (*Trypanosoma cruzi* or a *Leishmania* species). This heterologous mean is used to compute the 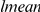 as above. As this value is considered to be more uncertain than a value measured in *T. brucei*, the 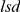 is calculated so that the upper or lower limit of the 95% confidence interval equals the upper or lower limit of the 95% confidence interval of all *T. brucei* enzyme retrieved from BRENDA (if the heterologous mean is higher than the mean calculated from all *T. brucei* enzyme retrieved from BRENDA, then the upper limit is used, otherwise, the lower limit is used). However, if the 95% confidence interval calculated is bigger than 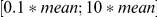 then 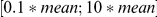 is used to calculate 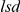.

The 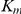 values of PGAM used in the published version of the model were measured in the presence of cobalt, as this was believed to be the cofactor used by the enzyme. However, the nature of the metallic cofactor used by this enzyme has recently been questioned: Fuad et al. (2011) [33] have shown that the concentration of cobalt is too small to be relevant *in vivo*. For this reason, the 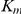 values of PGAM were set according to the earlier measurements done by Chevalier et al. (2000) [27]: 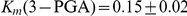 mM and 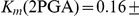
0.03 mM.

#### Keq

The equilibrium constants, 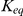, can be calculated from the Gibbs free energy of a reaction, 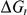, using equation 5:
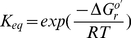
(5)

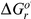
 is expressed in J/mol and can be positive, negative or null. Therefore, we assumed that our uncertainty about the exact value of 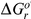 can be described by a normal distribution. As a consequence, according to equation 5, the plausible values of the equilibrium constant will be log-normally distributed.

As the equilibrium constant does not depend on the organism (assuming constant temperature, pH and ionic strength), the mean and standard-deviation of the distribution can be calculated from the various values reported in the literature (see supplementary text S3). When only one published value could be found, the standard deviation was calculated using the mean relative standard deviation of the other equilibrium constants in the model as described above.

#### Vmax

The maximum rate of the reactions (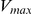) can only have positive values. The 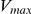 values are linked to the equilibrium constant and 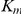 values by the Haldane equation. Therefore, we assume again that our uncertainty about them can best be described by a log-normal distribution.

For each 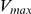, the mean and standard deviation of the log-normal distribution must be defined (respectively 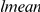 and 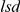). When the 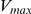 had been measured, the 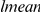 and 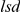 were calculated the same way as for the 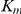 values. When no information was available, the 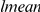 was set using the value fitted by Alberts et al. [10] for the fixed-parameter model. In these cases, the 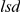 was then set so that the upper limit of the confidence interval (95%) is 4000 nmol/min/mg protein (the largest 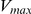 in the model is 2862 nmol/min/mg protein for phosphoglycerate kinase [10]).

If the 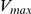 was measured in the reverse direction (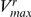), 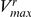 is sampled. 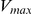 is then calculated from the sampled 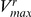, 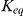 and 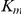 values using the Haldane equation. The value used for phosphoglycerate kinase is calculated from the measured 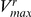. However, as two of the 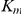 values were not measured and therefore have large standard deviations, sampling this 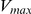 from the 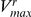, 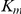 and 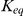 would result in sampling values much larger than 4000 nmol/min/mg of proteins. Therefore, the phosphoglycerate kinase 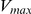 was sampled using the calculated value as a mean, and the standard deviation was calculated so that the upper limit of the confidence interval (95%) is 4000 nmol/min/mg protein.

The 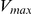 of GAPDH reported in the literature [10] was measured in crude extracts where a non-identified metabolite seems to inhibit it [25]. The inhibition factor (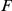) was estimated by Misset et al. [25] as about three-fold and was sampled separately in our study. As 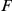 needs to be higher than 1, it was sampled using a log-normal distribution (with 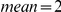 and 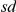 calculated so that the upper limit of the confidence interval (95%) is 3.5) to which 1 is added. The 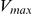 of GAPDH in the model is then multiplied by this sampled inhibition factor.

The glucose transporter 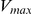 was set according to the measurements of Seyfang et al. [34]. Using the 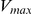 at 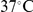 and the activation energy they measured, we estimated the 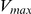 of glucose transporter at 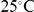 to 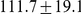 nmol/min/mg of protein. Note that this value is close to the fitted value used in [10] (108.9 nmol/min/mg of protein).

#### Transport reactions

The model includes several transport reactions. Among them, only the transport rates across the cytosolic membrane have been measured. The transport rates across the glycosomal membrane have not been characterised and are currently modelled using mass action kinetics (i.e., as non-saturable, non-enzymatic reactions) to maintain maximal compatibility with the published model [10], [11]. The corresponding parameters have not been measured. The equilibrium constant of these transport reactions is assumed to be 1, so that only one kinetic parameter is required per transport reaction.

No information is available about the uncertainty of these parameters. As these parameters are strictly positive, they are sampled using a log-normal distribution as are 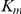 and 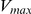 values. The means are set to the minimum value so that the reaction will be within 5% of equilibrium (using the mean values for all the other parameters). The standard deviation is calculated so that the upper limit of the confidence interval (95%) is equal to 100 times the mean to allow a large exploration of the parameter space.

#### Specific cases

Bakker et al. [7] and the following versions of the model included adenylate kinase implicitly, considering this reaction to be at equilibrium. We modelled adenylate kinase using mass action kinetics, with two rate constants 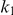 and 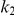. As only the equilibrium constant of this reaction is known, 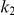 is sampled using the same methods as for the parameters of the transport reactions. 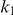 is then calculated from 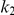 and the sampled equilibrium constant: 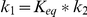.

ATP utilization is modelled using mass action kinetics with a single rate constant. As this reaction represents all of the cytosolic reactions that consume ATP and are not explicitly included in the model, the rate constant of this reaction is unknown. As for glycosomal transport reactions, this parameter was sampled according to a log-normal distribution. The mean used is the value fitted by Bakker et al. [7]. The standard deviation is calculated so that the upper limit of the 95% confidence interval equals 2 times the mean.

The glucose transport across the cytosolic membrane is assumed to be symmetric [7] based on experimental evidence [35]. Moreover, it exhibits a trans-acceleration phenomenon [36] which is quantify by a parameter, 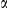 in the underlying model of the transporter kinetics. As this parameter varies between 0 and 1, it was sampled using a logit-normal distribution. The estimated value from Bakker et al. [7] was used as a mean. The standard deviation was arbitrarily set so that the upper limit of the 95% confidence interval is the mean +20%.

### Parameter sampling

All parameters were sampled using the MT19937 random number generator of Makoto Matsumoto and Takuji Nishimura [37] implemented in the GNU Scientific Library (GSL) [38]. The random numbers where then transformed to follow their assumed probability distribution using the random number distribution function implemented in the GSL library.

### Steady-state calculations

The steady states were calculated using the SOSlib library [39]. Steady-state is considered if the mean+standard deviation of the rates of change of all metabolite concentrations is lower than a user-defined parameter 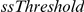 of SOSlib. The initial conditions were set using the steady-state concentrations calculated using the mean values of all parameters. For any sampled model, it is assumed that steady state should be reached within 300 minutes of simulated time (steady state detection threshold 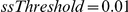, parameter 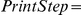
1 per simulated minute). We checked that the steady-state calculations give similar results in COPASI [40] and PySCeS [41] using their default parameters. We also verified that the parameter sets that do not allow the model to reach steady state in these conditions show accumulation of individual metabolites beyond reasonable concentrations (hundreds or thousands of millimol per liter, see Results and Fig. 3).

### Control coefficients

The control coefficients were computed using the methodology described by Bakker et al. [8]. The computation of control coefficients requires more precise steady-states calculations. Therefore, the parameters of SOSlib were set to: maximal time 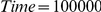 minutes and the threshold 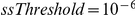.

## Supporting Information

Dataset S1 **Fixed-parameter model (sbml file).**
(XML)

Figure S1 **Examples of simulations of models unable to reach steady-state (within 1000 simulated minutes).** (A) Simulation of pyruvate concentration in a model unable to reach steady-state because of pyruvate accumulation. Models of this type will never reach steady-state. (B) Simulation of glycosomal 3-PGA concentration in a model unable to reach steady-state because of 3-PGA accumulation. Models of this type will eventually reach steady-state, but at extremely high concentrations of 3-PGA.
(TIFF)

Text S1 **Distributions of the sampled parameters.**
(PDF)

Text S2 **Distributions of the steady-state concentrations of the metabolites in mmol/l.**
(PDF)

Text S3 **Sources used for the calculation of the equilibrium constants mean and standard deviations.**
(PDF)
