# Supplementary figures and images for: Dynamic Modelling under Uncertainty: The Case of Trypanosoma brucei Energy Metabolism
Source: PLoS Comput Biol. 2012 Jan 19;8(1):e1002352. doi: 10.1371/journal.pcbi.1002352 (PMC3269904; doi:10.1371/journal.pcbi.1002352)

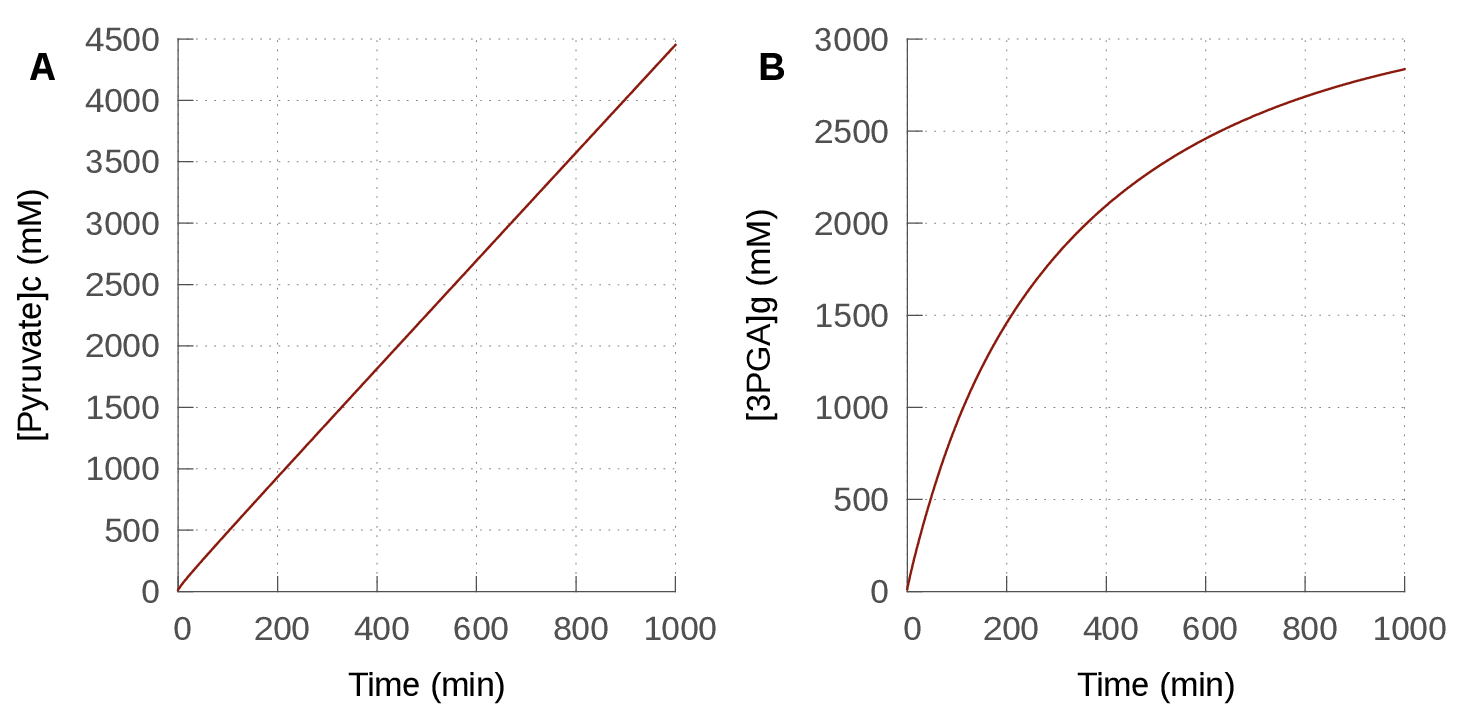

Supplement: Figure S1 — Examples of simulations of models unable to reach steady-state (within 1000 simulated minutes). (A) Simulation of pyruvate concentration in a model unable to reach steady-state because of pyruvate accumulation. Models of this type will never reach steady-state. (B) Simulation of glycosomal 3-PGA concentration in a model unable to reach steady-state because of 3-PGA accumulation. Models of this type will eventually reach steady-state, but at extremely high concentrations of 3-PGA. (TIFF) [file pcbi.1002352.s002.tif]
