# Supplementary material for: Dynamic Modelling under Uncertainty: The Case of Trypanosoma brucei Energy Metabolism
Source: PLoS Comput Biol. 2012 Jan 19;8(1):e1002352. doi: 10.1371/journal.pcbi.1002352 (PMC3269904; doi:10.1371/journal.pcbi.1002352)
Supplement: Text S1 — Distributions of the sampled parameters. (PDF) [file pcbi.1002352.s003.pdf]

Distributions of the sampled parameters. Parameter names followed by a * are shown log-scaled. The black line is the value used in the fixed-parameter model. List of abbreviations:

Reactions	
_3PGAT_g	glycosomal 3-phosphoglycerate transport
AK_c	cytosolic adenylate kinase
AK_g	glycosomal adenylate kinase
ALD_g	glycosomal aldolase
ATPu_c	cytosolic ATP utilization
ENO_c	cytosolic enolase
G3PDH_g	glycosomal glycerol 3-phosphate dehydrogenase
GAPDH_g	glycosomal glyceraldehyde phosphate dehydrogenase
GDA_g	glycerol 3-phosphate/dihydroxyacetone phosphate antiporter
GK_g	glycosomal glycerol kinase
GlcT_c	cytosolic glucose transport
GlcT_g	glycosomal glucose transport
GPO_c	cytosolic glycerol 3-phosphate oxidase
HXK_g	glycosomal hexokinase
PFK_g	glycosomal phosphofructose kinase
PGI_g	glycosomal phosphoglucose isomerase
PGK_g	glycosomal phosphoglycerate kinase
PGAM_c	cytosolic phosphoglycerate mutase
PYK_c	cytosolic pyruvate kinase
PyrT_c	cytosolic pyruvate transport
TPI_g	glycosomal triosephosphate isomerase
**Metabolites**	
13BPGA	1,3-biphosphoglycerate
2PGA	2-phosphoglycerate
3PGA	3-phosphoglycerate
ADP	adenosine diphosphate
AMP	adenosine monophosphate
ATP	adenosine triphosphate
DHAP	dihydroxyacetone phosphate
Fru16BP	fructose 1,6-biphosphate
Fru6P	fructose 6-phosphate
GA3P	glyceraldehyde 3-phosphate
Glc	glucose
Glc6P	glucose 6-phosphate
Gly	glycerol
Gly3P	glycerol 3-phosphate
NAD	nicotinamide adenine dinucleotide (oxidized form)
NADH	nicotinamide adenine dinucleotide (reduced form)
PEP	phosphoenolpyruvate
Pyr	pyruvate

Units:

Vmax	nmol/min/mg of proteins
Km, Ki	mmol/l
Keq	no unit except for aldolase: mmol/l
3PGAT_g_k	μl/min/mg of proteins
AK_c_k1	μl^2/nmol/min/mg of proteins
AK_c_k2	μl^2/nmol/min/mg of proteins
AK_g_k1	μl^2/nmol/min/mg of proteins
AK_g_k2	μl^2/nmol/min/mg of proteins
ATPu_c_k	nmol/min/mg of proteins
GDA_g_k	μl^2/nmol/min/mg of proteins
GlcT_g_k	μl/min/mg of proteins
GlcT_c_alpha	no unit
PYK_c_n	no unit

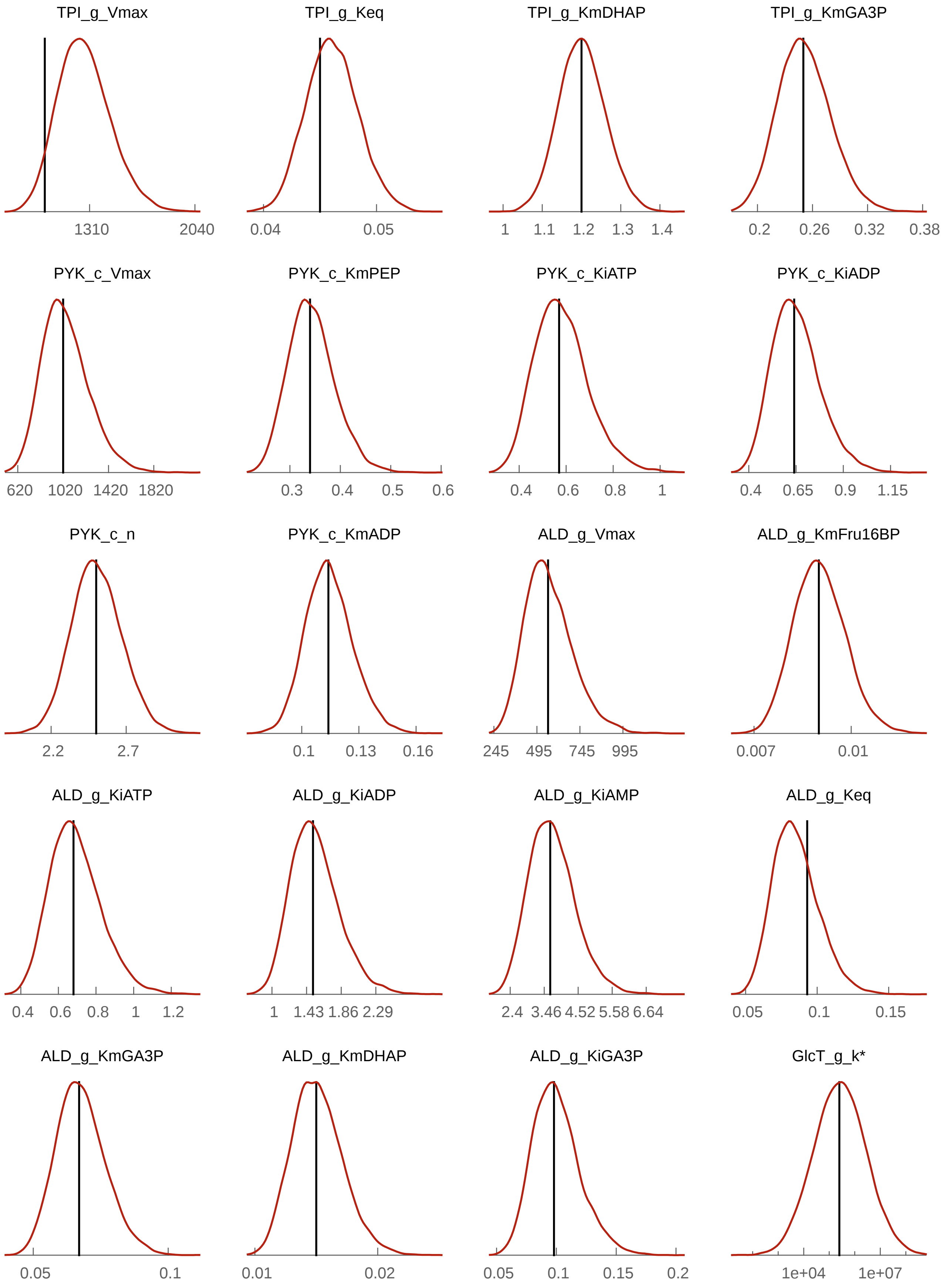
TPI_g_Vmax
1310
2040
TPI_g_Keq
0.04
0.05
TPI_g_KmDHAP
1
1.1
1.2
1.3
1.4
TPI_g_KmGA3P
0.2
0.26
0.32
0.38
PYK_c_Vmax
620
1020
1420
1820
PYK_c_KmPEP
0.3
0.4
0.5
0.6
PYK_c_KiATP
0.4
0.6
0.8
1
PYK_c_KiADP
0.4
0.65
0.9
1.15
PYK_c_n
2.2
2.7
PYK_c_KmADP
0.1
0.13
0.16
ALD_g_Vmax
245
495
745
995
ALD_g_KmFru16BP
0.007
0.01
ALD_g_KiATP
0.4
0.6
0.8
1
1.2
ALD_g_KiADP
1
1.43
1.86
2.29
ALD_g_KiAMP
2.4
3.46
4.52
5.58
6.64
ALD_g_Keq
0.05
0.1
0.15
ALD_g_KmGA3P
0.05
0.1
ALD_g_KmDHAP
0.01
0.02
ALD_g_KiGA3P
0.05
0.1
0.15
0.2
GlcT_g_k*
1e+04
1e+07

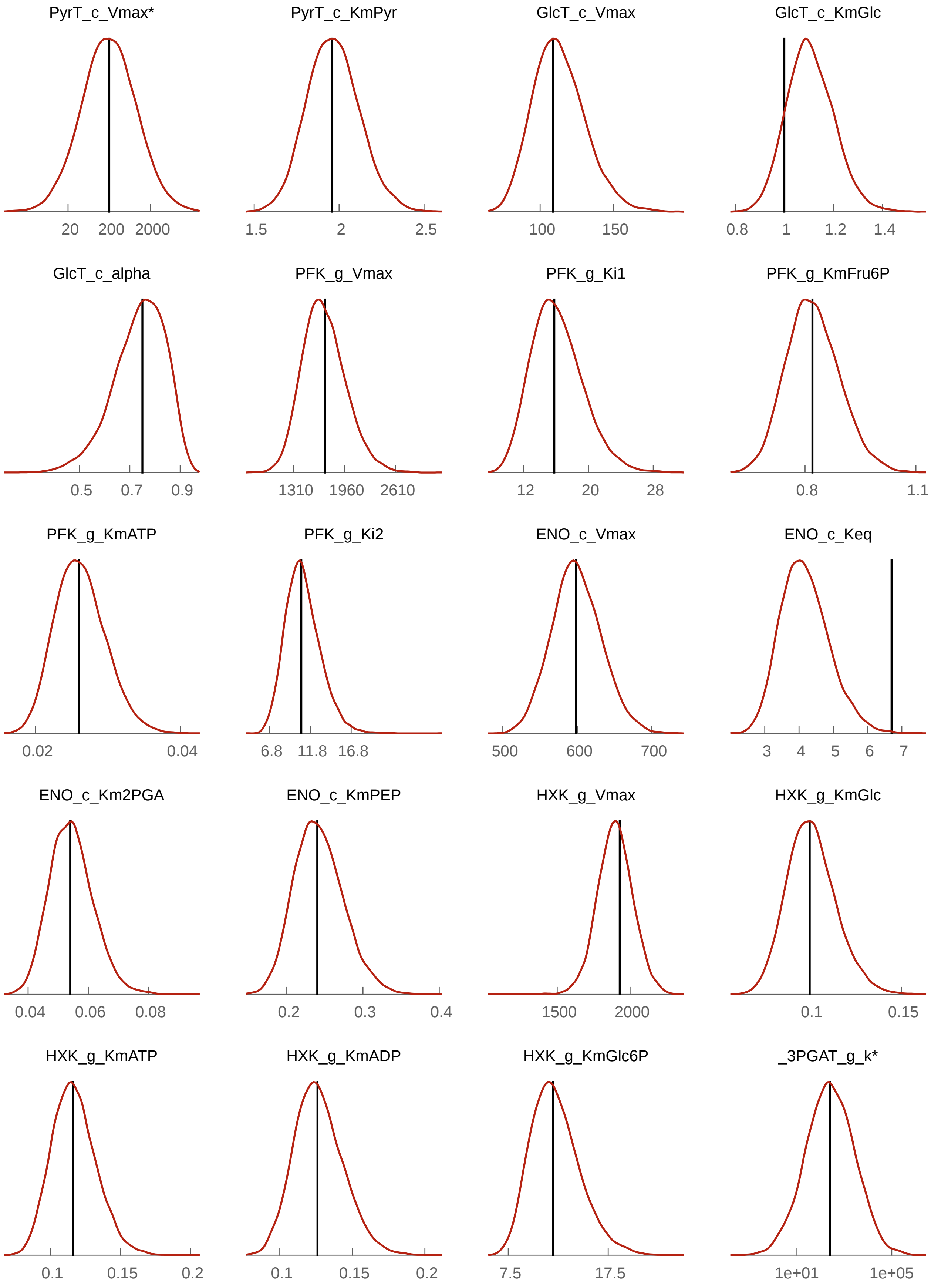
PyrT_c_Vmax*
20 200 2000
PyrT_c_KmPyr
1.5 2 2.5
GlcT_c_Vmax
100 150
GlcT_c_KmGlc
0.8 1 1.2 1.4
GlcT_c_alpha
0.5 0.7 0.9
PFK_g_Vmax
1310 1960 2610
PFK_g_Ki1
12 20 28
PFK_g_KmFru6P
0.8 1.1
PFK_g_KmATP
0.02 0.04
PFK_g_Ki2
6.8 11.8 16.8
ENO_c_Vmax
500 600 700
ENO_c_Keq
3 4 5 6 7
ENO_c_Km2PGA
0.04 0.06 0.08
ENO_c_KmPEP
0.2 0.3 0.4
HXK_g_Vmax
1500 2000
HXK_g_KmGlc
0.1 0.15
HXK_g_KmATP
0.1 0.15 0.2
HXK_g_KmADP
0.1 0.15 0.2
HXK_g_KmGlc6P
7.5 17.5
_3PGAT_g_k*
1e+01 1e+05

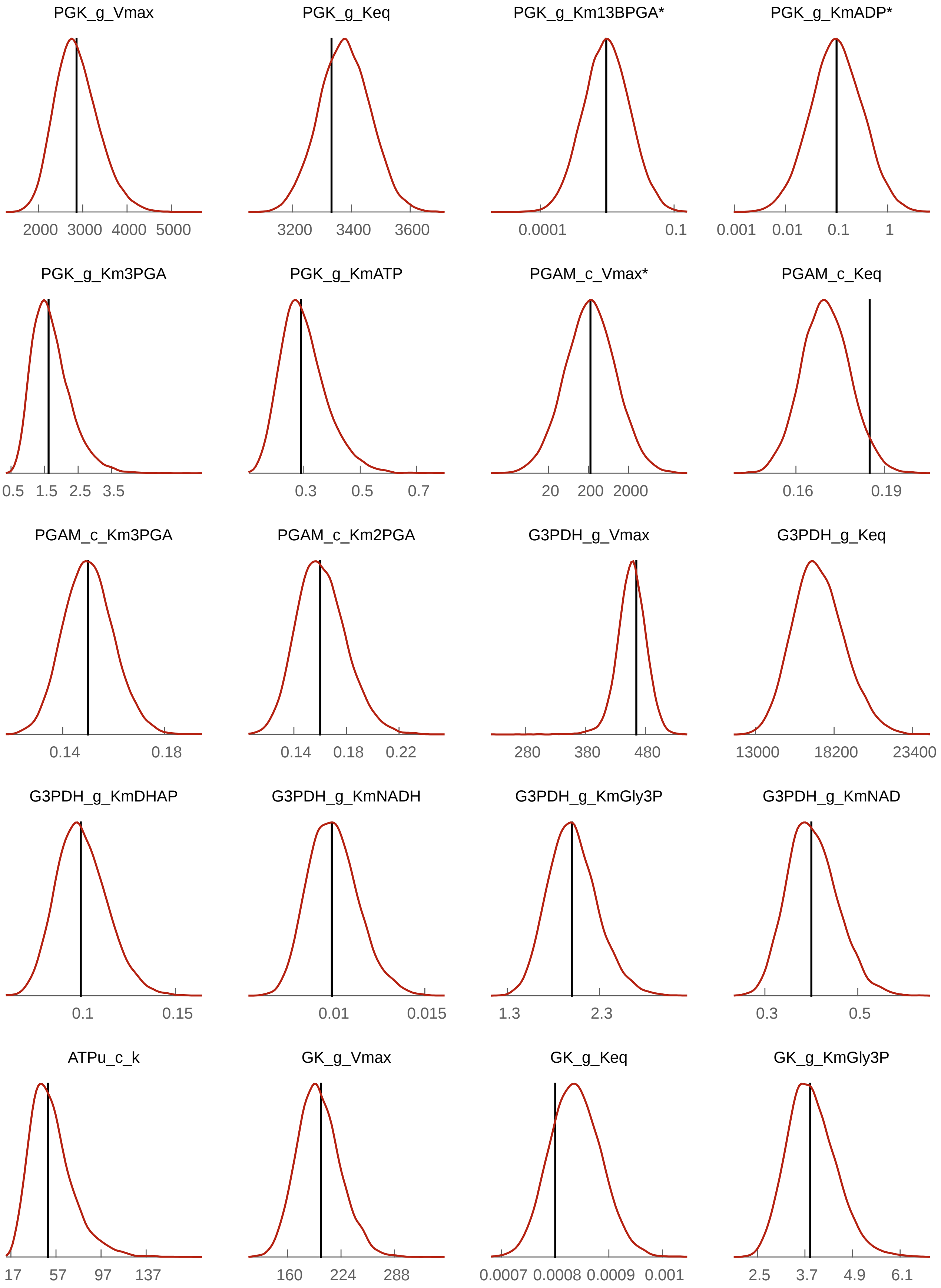
PGK_g_Vmax
2000 3000 4000 5000
PGK_g_Keq
3200 3400 3600
PGK_g_Km13BPGA*
0.0001 0.1
PGK_g_KmADP*
0.001 0.01 0.1 1
PGK_g_Km3PGA
0.5 1.5 2.5 3.5
PGK_g_KmATP
0.3 0.5 0.7
PGAM_c_Vmax*
20 200 2000
PGAM_c_Keq
0.16 0.19
PGAM_c_Km3PGA
0.14 0.18
PGAM_c_Km2PGA
0.14 0.18 0.22
G3PDH_g_Vmax
280 380 480
G3PDH_g_Keq
13000 18200 23400
G3PDH_g_KmDHAP
0.1 0.15
G3PDH_g_KmNADH
0.01 0.015
G3PDH_g_KmGly3P
1.3 2.3
G3PDH_g_KmNAD
0.3 0.5
ATPu_c_k
17 57 97 137
GK_g_Vmax
160 224 288
GK_g_Keq
0.0007 0.0008 0.0009 0.001
GK_g_KmGly3P
2.5 3.7 4.9 6.1

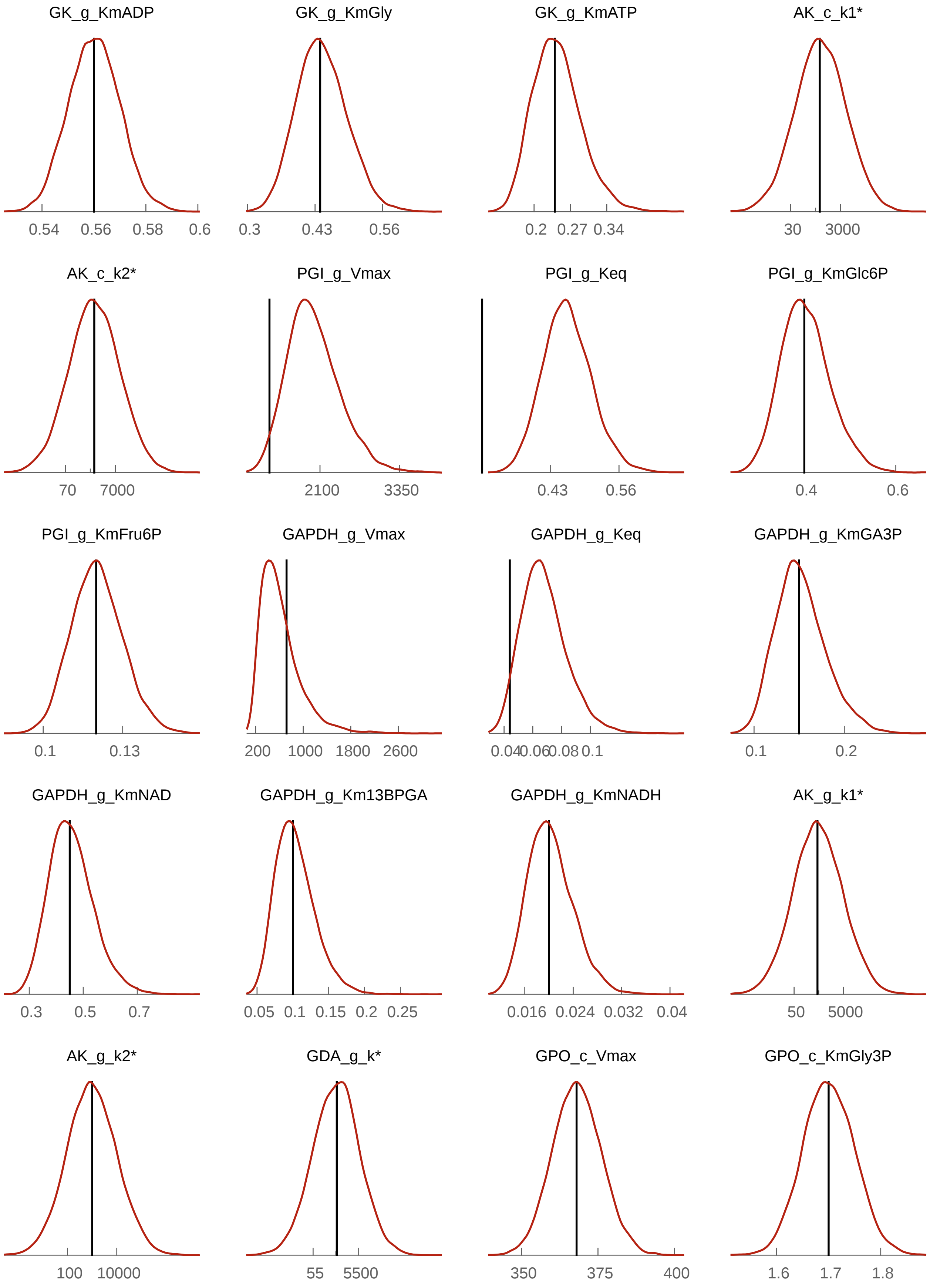
GK_g_KmADP
0.54 0.56 0.58 0.6
GK_g_KmGly
0.3 0.43 0.56
GK_g_KmATP
0.2 0.27 0.34
AK_c_k1*
30 3000
AK_c_k2*
70 7000
PGI_g_Vmax
2100 3350
PGI_g_Keq
0.43 0.56
PGI_g_KmGlc6P
0.4 0.6
PGI_g_KmFru6P
0.1 0.13
GAPDH_g_Vmax
200 1000 1800 2600
GAPDH_g_Keq
0.04 0.06 0.08 0.1
GAPDH_g_KmGA3P
0.1 0.2
GAPDH_g_KmNAD
0.3 0.5 0.7
GAPDH_g_Km13BPGA
0.05 0.1 0.15 0.2 0.25
GAPDH_g_KmNADH
0.016 0.024 0.032 0.04
AK_g_k1*
50 5000
AK_g_k2*
100 10000
GDA_g_k*
55 5500
GPO_c_Vmax
350 375 400
GPO_c_KmGly3P
1.6 1.7 1.8
