# Supplementary material for: Dynamic Modelling under Uncertainty: The Case of Trypanosoma brucei Energy Metabolism
Source: PLoS Comput Biol. 2012 Jan 19;8(1):e1002352. doi: 10.1371/journal.pcbi.1002352 (PMC3269904; doi:10.1371/journal.pcbi.1002352)

Distributions of the steady-state concentrations of the metabolites in mmol/l.
Metabolite names followed by a * are shown log-scaled.

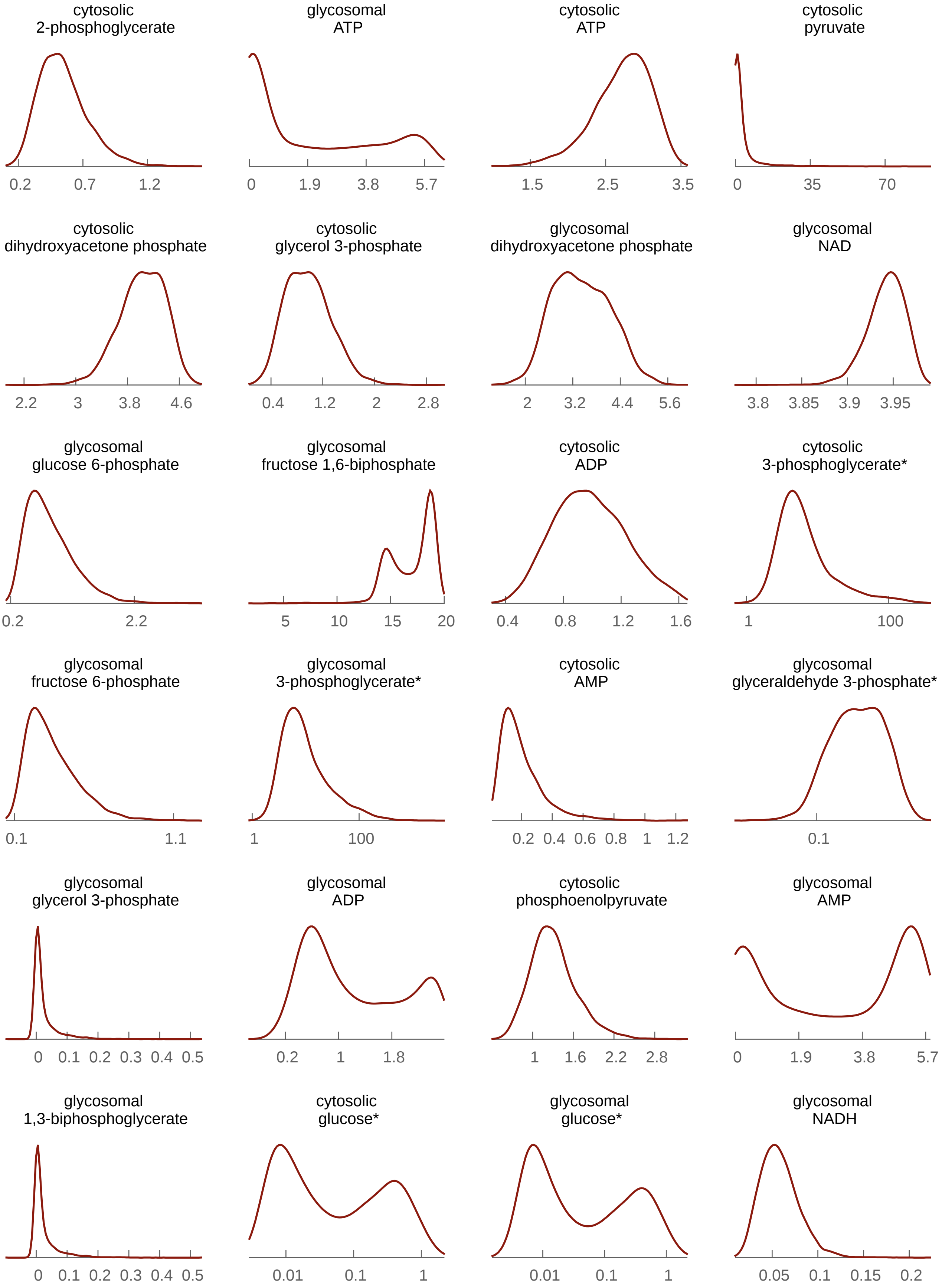

Supplement: Text S2 — Distributions of the steady-state concentrations of the metabolites in mmol/l. (PDF) [file pcbi.1002352.s004.pdf]
