# Supplementary material for: Dynamic Modelling under Uncertainty: The Case of Trypanosoma brucei Energy Metabolism
Source: PLoS Comput Biol. 2012 Jan 19;8(1):e1002352. doi: 10.1371/journal.pcbi.1002352 (PMC3269904; doi:10.1371/journal.pcbi.1002352)
Supplement: Text S3 — Sources used for the calculation of the equilibrium constants mean and standard deviations. (PDF) [file pcbi.1002352.s005.pdf]

Reaction	Sources	Mean	SEM
Adenylate kinase	[1]	0.48	0.015
Phosphoglucose isomerase	[2,3]	0.457	0.048
Aldolase	[2,3]	0.084	0.017
Triose phosphate isomerase	[2,3]	0.046	0.0023
Glyceraldehyde-3-phosphate dehydrogenase	[2,3],NIST(50COR/VEL_252)*	0.066	0.017
Glycerol-3-phosphate dehydrogenase	NIST(58YOU/PAC_45+, 37EUL/ADL2_42+, 49BAR_43)	17085	1782
Phosphoglycerate kinase	[4], NIST(70KRI/BUC_579)	3377	88
Glycerol kinase	[5,6]#, [7]	8.37e-4	4.8e-5
Phosphoglycerate mutase	[2, 3], NIST(49MEY/OES_1388, 59CHI/SUG_1391, 75GRI/CAR_1396)	0.17	0.0084
Enolase	[2,4,8], NIST(57WOL/BAL_1173)	4.17	0.75

Table 1. Sources used for the calculation of the equilibrium constants mean and standard deviations. The references retrieve from the NIST Standard Reference Database [9] are specified as NIST(id). SD=standard deviation of the values from all papers (for adenylate kinase the series of values given in [1] is used). *=values corrected for pH as in [10]. +=values corrected for pH using a series of values at different pH. #=calculated from measured Km and Vmax using the Haldane equation.

References

1. Atkinson MR, Burton RM, Morton RK (1961) Equilibrium constant of phosphoryl transfer from adenosine triphosphate to galactose in the presence of galactokinase. Biochem J 78: 813–820.

2. Voet D, Voet JG, Pratt CW (1999) Fundamentals of biochemistry. Wiley.

3. Lehninger AL, Nelson DL, Cox MM (2005) Principles of biochemistry, volume 1. Worth.

4. Bergmeyer HU (1974) Methods of enzymatic analysis. Verlag Chemie.

5. Kralova I, Rigden DJ, Opperdoes FR, Michels PAM (2000) Glycerol kinase of *Trypanosoma brucei.* cloning, molecular characterization and mutagenesis. European Journal of Biochemistry 267: 2323–2333.

6. Janson CA, Cleland WW (1974) The kinetic mechanism of glycerokinase. Journal of Biological Chemistry 249: 2562 –2566.

7. Burton K (1961) Biochemists' Handbook. In: Biochemists' Handbook, London: E. & F. N. Spon Ltd. C. long. edition, p. 94.

8. Meyerhof O, Green H (1949) Synthetic action of phosphatase; equilibria of biological esters. The Journal of Biological Chemistry 178: 655–667.

9. Goldberg RN, Tewari YB, Bhat TN (2004) Thermodynamics of enzyme-catalyzed reactions – a database for quantitative biochemistry. Bioinformatics 20: 2874 –2877.

10. Alberty RA (1998) Calculation of standard transformed Gibbs energies and standard transformed enthalpies of biochemical reactants. Archives of Biochemistry and Biophysics 353: 116–130.
